# Surgical and Non-Surgical Approach for Tear Trough Correction: Fat Repositioning Versus Hyaluronic Acid Fillers

**DOI:** 10.3390/jpm14111096

**Published:** 2024-11-06

**Authors:** Stylianos Christodoulou, Argyrios Tzamalis, Ioannis Tsinopoulos, Nikolaos Ziakas

**Affiliations:** 1 Postgraduate Master Program “Ocular Surgery”, Aristotle University of Thessaloniki, 541 24 Thessaloniki, Greece; atzamalis@auth.gr (A.T.); itsinop@auth.gr (I.T.); ngziakas@auth.gr (N.Z.); 2 2nd Department of Ophthalmology, Aristotle University of Thessaloniki, Papageorgiou General Hospital, 564 29 Thessaloniki, Greece

**Keywords:** tear trough, fat reposition, lower blepharoplasty, hyaluronic acid, HA fillers

## Abstract

**Objective**: This paper compares two popular techniques for tear trough correction—fat repositioning and hyaluronic acid (HA) fillers—highlighting their efficacy, safety profiles, patient satisfaction, and associated complications. **Methods**: A narrative review of 20 studies comparing fat repositioning and HA fillers was conducted, focusing on parameters such as duration of results, volume restoration, complication rates, and patient satisfaction. **Results**: Fat repositioning provides long-lasting results but carries higher surgical risks compared with HA fillers. The transconjunctival approach is suitable for patients with minimal skin excess. The supraperiosteal plane allows for a quicker procedure and, despite postoperative edema and temporary irregular contouring, shows no difference in final cosmetic outcomes compared with other planes. Internal fixation reduces the risk of fat relapse and skin scarring but carries the risk of suboptimal positioning. HA fillers offer immediate, minimally invasive results but require periodic maintenance. The use of a cannula reduces the risk of vascular occlusion. Combining a high G’ filler for the midface with a low G’ with low hydrophilicity for the tear trough reduces the amount of filler needed and prolongs the results. Both surgical and non-surgical methods are effective, depending on patient needs and anatomical considerations. **Conclusions**: Fat repositioning is ideal for patients seeking long-term correction and are willing to undergo surgery, while HA fillers suit those preferring non-invasive treatments with customizable, short-term effects. Both techniques have pros and cons that must be matched to patient goals and conditions.

## 1. Introduction

The tear trough, a subtle depression running from the inner corner of the eye to the mid-cheek, plays a crucial role in facial aesthetics and is a focal point in cosmetic ophthalmology [1]. It acts as a transition zone between the lower eyelid and the cheek, defined by thin skin and minimal subcutaneous tissue [2]. The tear trough’s appearance is influenced by a complex interplay of anatomical structures, including ligaments, fat pads, and vascular elements. The lower eyelid’s anatomy—comprising layers such as skin, the orbicularis oculi muscle, orbital septum, and suborbicularis oculi fat (SOOF)—contributes significantly to the tear trough’s contour [3,4]. Age-related changes, such as skin laxity, muscle weakening, and fat herniation, can accentuate tear trough deformities, making them more pronounced and contributing to a fatigued or aged appearance.

The midface anatomy also impacts tear trough deformities, with fat compartments like the malar and submalar pads playing a key role in facial fullness and support [5]. With age, the descent and atrophy of these fat pads lead to midface deflation, further emphasizing tear trough hollowness. Understanding the vascular anatomy of the tear trough region is essential for safe cosmetic interventions, as this area includes critical arteries and veins that support facial structures. Key arteries include the supratrochlear, infraorbital, and angular arteries, which interconnect to form a complex vascular network. The venous system, including the supratrochlear and infraorbital veins, ensures effective drainage and maintains venous circulation, highlighting the importance of anatomical knowledge for successful cosmetic procedures [6,7].

Aging brings significant anatomical changes to the tear trough and midface region, contributing to the development of tear trough deformities. The loss of soft tissue volume, midface descent, and weakening of ligamentous support structures lead to more prominent tear troughs. Additionally, age-related skin changes such as decreased collagen production and thinning of the skin make underlying structures more visible, further highlighting tear trough hollowness. Bone resorption and fat redistribution also alter the structural support of the midface, accentuating tear troughs. These changes manifest differently across various age groups: younger adults may experience congenital or developmental issues, middle-aged individuals face gradual subcutaneous fat loss and skin thinning, and older adults often encounter bone resorption and fat herniation [8].

Accurate assessment of tear trough deformities is crucial for effective treatment, with classification systems like the Hirmand Classification and Barton Grading System offering standardized approaches to evaluate severity [9]. These classifications guide treatment decisions, ranging from minimally invasive hyaluronic acid (HA) fillers to more permanent surgical interventions like fat repositioning. HA fillers provide immediate, customizable results but require maintenance, while fat repositioning offers longer-lasting outcomes through surgical fat transposition. Each method has its own risks and benefits, making evidence-based decision making vital for tailoring treatment to individual patient needs [10,11]. Ultimately, tear trough deformities can significantly impact quality of life, affecting self-esteem, social interactions, and professional perceptions, underscoring the importance of effective, patient-specific cosmetic interventions [12].

Our study is important because it provides evidence-based guidance on choosing between fat repositioning and hyaluronic acid (HA) fillers for tear trough correction, a common aesthetic concern that impacts both appearance and self-esteem. Despite the widespread use of both non-invasive (HA fillers) and invasive (fat repositioning) techniques, the existing literature predominantly focuses on single methods, without offering comprehensive comparisons. Advances in both techniques have increased the need for a clearer evaluation, especially given conflicting results regarding complications and long-term outcomes. By synthesizing the available evidence, this review aims to give clinicians more informed, individualized guidance on selecting the most appropriate treatment for tear trough correction.

## 2. Materials and Methods

### 2.1. Search Strategy

A comprehensive search strategy was developed to identify relevant studies addressing the treatment of tear trough deformity. The research question was framed using the population, intervention, comparison, outcome (PICO) framework, focusing on patients seeking cosmetic treatment for tear trough deformity. Two primary interventions were investigated: fat repositioning and hyaluronic acid (HA) filler injections, chosen for their widespread use in periorbital rejuvenation. The outcomes of interest included efficacy, safety, patient satisfaction, and treatment longevity.

The databases used for the search were PubMed, Cochrane, Scopus, and Web of Science. The search was conducted using a combination of controlled vocabulary (MeSH terms) and free-text terms to ensure a thorough capture of relevant literature. The following search terms were employed in a single line:

(“tear trough” OR “periorbital rejuvenation”) AND (fat OR fillers OR “HA fillers” OR “Hyaluronic acid” OR “lower blepharoplasty” OR injectable).

Search filters were applied to limit the results to English-language studies published before 27 March 2024. Additionally, only studies that involved human subjects were included.

### 2.2. Inclusion and Exclusion Criteria

Inclusion criteria:

- Study design: randomized controlled trials, cohort studies, cross-sectional studies
- English language
- Tear trough deformity treated with fat reposition or HA fillers
- Studies that include outcomes related to efficacy and/or safety, patient satisfaction, longevity of the mentioned interventions

Exclusion criteria:

- Study designs: reviews (systematic or narrative), abstracts only, book chapters, letters, unfinished studies, case studies and reports
- Tear trough deformity treated with method other than fat reposition or HA fillers

## 3. Results

The initial search retrieved 370 records from PubMed, 23 from Cochrane, 1045 from Scopus, and 455 from Web of Science, totaling 1893 articles. After removing duplicates and screening titles and abstracts for relevance, 20 studies were selected for inclusion in this review. The final selection was based on relevance to the predefined research question and inclusion/exclusion criteria.

### 3.1. Fat Repositioning Techniques Studies

The studies described various surgical techniques to address tear trough deformity and lower eyelid aging, each offering unique benefits and complications. Liapakis et al. (2014) introduced a surgical procedure for tear trough correction, highlighting a subciliary incision with fat redistribution and lateral eye lift, achieving minimal complications and high patient satisfaction. Although some patients experienced edema, conjunctivitis (1/35), and scleral show (7/35), these were generally manageable, and most patients reported high satisfaction with the results [1]. Mohadjer and Holds (2006) proposed a technique with fat repositioning in an intra-suborbicularis oculi fat (intra-SOOF) plane, emphasizing minimal complications and high satisfaction, despite a few cases requiring revision due to ectropion or residual fat [2]. Majidian et al. used transconjunctival blepharoplasty with fat transposition above the orbicularis muscle, yielding good outcomes and minimal complications, with only minor bruising, swelling, and redness [3]. Gawdat et al. examined transconjunctival blepharoplasty with and without tear trough–orbicularis retaining ligament complex release, finding that the group with ORL release exhibited higher patient satisfaction and more significant improvements in tear trough deformity, although with a greater risk of bleeding and postoperative hematoma [4]. Cheng et al. (2024) have described supraperiosteal fat repositioning with midface lift in lower eyelid blepharoplasty, demonstrating favorable outcomes, high patient satisfaction, and minimal complications, despite a small percentage of patients requiring corrective procedures for ectropion [5]. Ding et al. (2022) presented an approach with intraoral fixation and redistribution of lower eyelid fat, showing high patient satisfaction and minimal complications, while providing an aesthetically pleasing result [6].

The “fan-shaped thin fat pedicles” technique, as presented by Cheng et al., utilizes transconjunctival incisions to reposition orbital fat. It involves fixation with mattress sutures to create durable lower lid contours [7]. This method showed significant improvement in tear trough depth and had minimal complications, such as only one case of infection, which resolved with antibiotics and suture removal. The technique’s unique approach ensures a stable outcome without bulges or suture failure. Chen et al. introduced a technique combining internal and external fixation during transconjunctival lower blepharoplasty. Fat pads are secured to the orbital rim using internal sutures, while external sutures are placed along the tear trough. The method proved effective in reducing tear trough deformities, with high patient satisfaction. However, complications included transient granulomas, postoperative swelling, eyelid numbness, and dry eye, all resolving spontaneously [8].

Chang et al. explored an internal fixation method called EZ-Tcon, which employs a 5-0 absorbable suture with three adjustable points for internal fixation during lower eyelid blepharoplasty. This method showed a lower rate of postoperative complications and higher aesthetic success rates than conventional external pull-out suture techniques. Notably, the internal fixation technique offered better patient satisfaction, earlier recovery, and reduced risk of long-lasting scars on the lower eyelid [9]. 

A summary of the key findings from the studies above is presented in Table 1. 

### 3.2. HA Filler Studies

Donath et al. (2010) used Restylane^®^ (Galderma, Lausanne, Switzerland) injections to rejuvenate the tear trough, employing a 3D imaging approach to assess volume changes. Bilateral injections were performed in the suborbicularis oculi muscle plane with a maximum of 0.5 mL of filler per tear trough [13]. Sharad (2020) published a study that involved the use of needle and cannula techniques to treat infraorbital hollow, using a low G’ HA Filler, Volbella^®^ (Allergan plc, Dublin, Ireland). The study employed a vertical supraperiosteal depot technique (VSDT) with needles for point injections, and blunt-tipped cannulas for retrograde placement along the tear trough region [14].

Diwan et al. (2020) utilized a cannula-based technique to inject Teosyal^®^ Redensity II (Teoxane Laboratories, Geneva, Switzerland) in the supra-periosteal plane. Filler volumes ranged from 0.2 to 0.6 mL per session [10]. Bernardini et al. (2021) aimed to realign the orbicularis retaining ligament (ORL) using soft tissue fillers. The treatment protocol involved injections to reposition the ligament’s orientation, potentially improving the appearance of the lid–cheek junction. The study suggested that filler augmentation could reorient the ORL and improve the appearance of the lid–cheek junction [11]. Vadera et al. (2021) compared the traditional 3-point tear trough technique using low G’ fillers with a lateral injection technique using high G’ fillers. The latter involved injections around the orbital wall, malar area, and zygoma, using small amounts for precise augmentation [12]. Berguiga and Galatoire (2017) assessed the safety and effectiveness of Teosyal^®^ Redensity II (Teoxane Laboratories, Geneva, Switzerland) using serial puncture and retrograde injection techniques with 30-gauge needles or cannulas. No serious adverse events occurred. Side effects included bruising (11%), swelling (12%), and redness (12%), with rare blue discoloration (2.6%) [15].

Hill III et al. (2015) examined Restylane^®^ (Galderma, Lausanne, Switzerland) and Perlane^®^ (Galderma, Lausanne, Switzerland) for volume enhancement in the tear trough and cheek areas. Perlane^®^ (Galderma, Lausanne, Switzerland) was used for cheek augmentation, while Restylane^®^ (Galderma, Lausanne, Switzerland) was used in the tear trough area with a retrograde fanning technique. The study found that direct tear trough injections had a more profound cosmetic impact compared with cheek augmentation alone. Despite expectations that cheek augmentation might indirectly improve the tear trough, direct treatment was found to be more effective. The study concluded that combining tear trough and cheek augmentation produced the most cosmetically appealing results [16]. Viana et al. (2011) used a serial puncture technique with a 30-gauge needle to place filler in the preperiosteal tissues below the orbital rim. The study demonstrated high patient satisfaction, with 88% showing cosmetic improvement in the tear trough area [17]. 

Table 2 provides an overview of the primary findings from the studies mentioned above.

## 4. Discussion

The choice between fat repositioning and HA fillers for tear trough correction depends heavily on patient-specific factors such as the severity of the tear trough deformity, desired duration of results, tolerance for surgical risks, and personal preferences regarding recovery time. Each technique presents distinct advantages and limitations that must be carefully considered.

### 4.1. Considerations for Fat Reposition Approach

Among the various surgical methods, surgical fat pad removal offers a direct approach but may worsen the hollow appearance and cause lid retraction [2]. Autologous fat grafting, a minimally invasive procedure, uses the patient’s own fat to rejuvenate the tear trough, may not be long lasting due to fat resorption. Conversely, fat repositioning stands out for its ability to deliver long-lasting and natural-looking outcomes by repositioning fat from surrounding areas, albeit requiring surgical intervention and posing potential risks such as swelling, bruising, and asymmetry. Despite these considerations, fat repositioning offers a promising solution for correcting tear trough deformity, addressing both volume augmentation and the descent of facial tissues, ultimately achieving a smoother facial contour. The transconjunctival method through the fornix (transforniceal) is appropriate for individuals experiencing lower fat pad protrusion and limited skin excess, whereas the transcutaneous technique through the subciliary path is specifically for candidates necessitating skin removal [1,5].

The preference between the subperiosteal and supraperiosteal planes for fat transposition is still a topic of debate [5]. Fat transposition via supraperiosteal plane seems to be quicker, but it may lead to more postoperative edema, bruising and short-term uneven contour. The final cosmetic outcome shows no difference compared with that achieved via subperiosteal plane. Blunt dissection prevents transection of angular vessel bleeding and it seems that the blood supply within the supraperiosteal plane could enhance the extended viability of fat pedicles. Moreover, careful dissection is advised to a maximum level of 10 mm below the orbital rim, in order to reduce the risk of intraorbital nerve injury [18].

Surgical intervention involving the release of the ORL to allow redistribution of orbital fat has demonstrated efficacy as a treatment option. Fixation is crucial for the scar formation and stability of repositioned fat. External fixation allows for easy placement at the lowest position but may be less secure. In contrast, internal fixation reduces the risk of fat relapse but involves a steep learning curve due to the narrow operation field and the possibility of incorrect positioning [9].

### 4.2. Considerations for the HA Filler Approach

Nonsurgical filler injections provide immediate improvement but necessitate regular maintenance injections for sustained results. Reported complications are ecchymosis and swelling, pain and erythema at the injection site, irregularities, and the Tyndall effect. On the other hand, HA appears to be a quick, efficient, and safe nonsurgical option for individuals with decent skin quality and reasonable tear trough deformity. The use of a cannula seems to have less risk of aesthetic or vascular complications, while combining low and high G’ fillers leads to a reduced amount of filler required, a longer lasting result, less frequent repetitions and less complications [12]. When using a needle, a 30-gauge needle appears to be safe for vertical supraperiosteal techniques along the inferior area of the orbit, medially and laterally, while micro-aliquots are recommended for the medial part of the tear trough [14]. Regarding the duration of the HA filler, it has been reported to last from 6 months to 2 years [1,2].

### 4.3. Fat Reposition vs. HA Fillers

Comparing hyaluronic acid (HA) fillers and fat repositioning for tear trough deformity requires a focus on efficacy, duration of results, patient satisfaction, and complications. Addressing the limitations of both techniques is essential. Fat repositioning may be more appropriate for patients with significant tear trough deformities (Barton Grade III or Hirmand Class III) or excess skin, particularly in older individuals seeking long-term, durable results. In contrast, HA fillers may be better suited for patients with milder tear trough concerns (Barton Grade I–II or Hirmand Class I–II), younger patients seeking non-invasive treatments, or those with limited recovery time [4,19].

Factors such as skin quality, elasticity, and anatomy—such as the depth of the tear trough or degree of lower eyelid hollowing—should also be considered, as these can impact the effectiveness and satisfaction of each method [14]. For example, in patients with pigmented skin but no tear trough hollowing, neither of the above techniques may be helpful. On the other hand, when there is herniation of fat pads, HA may provide limited results, and fat repositioning could be a better option [5].

Below is a comparative analysis drawn from the 20 studies included in this review.

#### 4.3.1. Efficacy

Fat repositioning: Surgical fat repositioning aims to achieve more lasting changes by redistributing existing fat. Techniques like transconjunctival blepharoplasty, intra-SOOF repositioning, and others, generally result in noticeable improvements with lower rates of recurrence. Studies note effective tear trough correction with minimal complications, demonstrating good surgical outcomes [1,2,7].

HA fillers: HA fillers have proven to be effective in addressing tear trough deformity, offering immediate volumetric improvement. Several studies report significant elevation of the tear trough with results lasting up to 15 months or more. Techniques such as Teosyal^®^ Redensity II (Teoxane Laboratories, Geneva, Switzerland) and Restylane^®^ (Galderma, Lausanne, Switzerland) have achieved high Global Aesthetic Improvement Scale (GAIS) scores and positive patient feedback [10,13,15].

#### 4.3.2. Duration

Fat repositioning: This technique is designed for more permanent outcomes. Results can last for several years due to the structural alterations. Surgical procedures involving fat redistribution aim for stable outcomes with a focus on durability [1].

HA fillers: HA fillers provide long-lasting results, with some studies suggesting effects lasting up to 23 months. High G’ fillers offer extended duration, with patient satisfaction correlating with the longevity of the results. Semi-cross-linked HA fillers have a reputation for lasting 1–2 years [11,12].

#### 4.3.3. Patient Satisfaction

Fat repositioning: Fat repositioning tends to result in high satisfaction levels, particularly due to its long-lasting results and improved lower eyelid aesthetics. Patients often appreciate the minimized scarring and natural-looking outcomes. However, some procedures may lead to temporary swelling or other mild side effects, impacting initial satisfaction [3].

HA fillers: Patient satisfaction tends to be high with HA fillers. Many studies report over 75% satisfaction rates, with some exceeding 90%. Outcomes are often rated as “exceptional” or “very improved.” Positive feedback stems from immediate results, minimal downtime, and the ability to correct minor irregularities over time [10].

#### 4.3.4. Complications

Fat repositioning: Complications can include transient edema, conjunctivitis, and, in some cases, long term complications like scarring, ectropion or scleral show [1]. However, these are generally manageable. Some fat repositioning techniques involve internal and external suturing, with risks of postoperative granulomas or mild numbness [8]. Techniques like intra-SOOF repositioning and internal fixation with 5-0 absorbable sutures aim to reduce complications while ensuring stable outcomes [2].

HA fillers: Common complications with HA fillers include mild and temporary effects like bruising, edema, and redness. Studies highlight the importance of proper technique to avoid complications such as overfilling or asymmetry. Long term complications like filler migration and granulomas are reported [10,11,12,13,14]. Although blindness from tear trough filler injections is rare, it is a documented complication in medical literature, particularly when injections are incorrectly placed. Proper technique and anatomical knowledge are crucial to minimizing this risk [20]. Injecting into the infraorbital artery creates a potential pathway to the ophthalmic artery, due to its connection to the supraorbital and supratrochlear vessels. Retrograde migration along these routes can embolize the ophthalmic artery, causing blindness [21]. Beleznay et al. identified 98 cases of filler-induced vision changes, with the glabella (38.8%), nasal area (25.5%), nasolabials (13.3%), and forehead (12.2%) being the highest-risk areas. Autologous fat was implicated in 47.9% of cases, followed by hyaluronic acid at 23.5% [22].

## 5. Conclusions

In summary, HA fillers offer immediate and moderately lasting results without surgery, making them a preferred choice for many patients due to their minimal invasiveness and ability to correct tear trough deformities without extensive recovery periods. They also allow for adjustments to achieve desired aesthetic outcomes. Conversely, fat repositioning, a surgical intervention, provides longer-lasting results and a more permanent solution to tear trough deformities, although with possible surgical risks and complications. Understanding the details of both procedures is essential, as each has its own benefits and limitations. This understanding is crucial for customizing treatment strategies to meet the unique needs and preferences of each patient. Additionally, the expertise of the doctor is pivotal in ensuring that the chosen approach is skillfully executed, leading to optimal outcomes tailored to the individual’s situation.

## 6. Limitations and Strengths

The limitations of our study include the absence of pregnant and breastfeeding participants, different injectors and surgeons, variations in filler rheology and gaps in follow-up. The strengths of the study are the strict inclusion and exclusion criteria, a robust sample size, a diverse population with different demographic backgrounds, multiple study designs, a variety of techniques, and the use of scales and photodocumentation for patient satisfaction and as credibility tools.

## 7. Future Directions: Addressing Research Gaps and Exploring Novel Technologies

Future research should explore the development of longer-lasting HA fillers or alternative materials that could reduce treatment frequency. Utilizing AI and machine learning to create personalized treatment protocols based on large databases could enhance efficacy and satisfaction. Long-term comparative studies on fat repositioning versus HA fillers could provide insights into their durability, cost-effectiveness, and patient preferences. Additionally, new delivery systems for HA fillers, like micro-cannulas with assisted imaging, could improve precision and reduce complications.

## Figures and Tables

**Table 1:** Fat repositioning techniques synopsis.

Study	Main Technique	Main Benefit
**[1]**	Subciliary incision and canthopexy	Middle face elevation Reduced risk of ectropion Reduced risk of facial palsy
**[2]**	Intra-SOOF plane	Increased survival rate of graft Easily accessible plane Office-based procedure
**[3]**	Transconjuctival incision above the orbicularis oculi muscle	Easy manipulation of fat pedicle Reduced risk of granulomas Increased survival rate of graft
**[4]**	ORL release	Better outcome Higher patient satisfaction
**[5]**	Supraperiosteal fat reposition	Transcutaneous approach for individuals with skin excess Tranfornicated approach for minimizing scars
**[6]**	Intraoral fixation	Less damage to orbicularis oculi muscle and lower risk for ectropion, especially for elderly patients
**[7]**	Fan-shaped pedicles with subperiosteal fixation	Tailored to individual anatomy—better outcomes and satisfaction
**[8]**	Combination of internal and external fixation in prezygomatic and premaxillary plane	Planes are anatomically predisected—less trauma is caused The septum remains intact Prevention of prelapse Guaranteed correct position of transposed fat
**[9]**	EZ-Tcon fixation	Combines the benefits of internal and external fixation (less scars, more stability of graft)
**[10]**	Internal fixation in the premaxillary space	Pleasing appearance Stability
**[11]**	Intranasal fixation with a casagrande needle	Significant reduction of scars Flexible anchoring
**[12]**	Internal fixation via the supraperiosteal plane	Enhanced survival of fat pedicle and stability

**Table 2:** HA filler techniques synopsis.

Study	Main Technique	Main Benefit
**[15]**	Quantify Augmentation and duration effect, using 30-gauge needle	Studying long term durability and effects of facial aging
**[16]**	Use of a 30-gauge needle (VSDT) and cannula (middle tear trough)	Less filler migration Longer durability Less bruising
**[17]**	Supraperiosteal cannula using HA 15 mg/dL	No major complications High satisfaction (75%)
**[18]**	High and low G’ HA fillers for ORL reorientation	Better aesthetic outcome
**[14]**	High G’ HA fillers	Less incidence of Tyndall effect Duration >1.5 years Less complications
**[19]**	Use of a 30-gauge needle and cannula with low G’ HA filler	Less absorption of water Less risk of uncontrolled swelling/malar oedema
**[13]**	Midface augmentation	Better aesthetic outcome No complications
**[20]**	Use of a 30-gauge needle serial puncture (0.1 mL each point), medial tear trough treated first	Less skin irregularities Maximizes tissue coverage Minimizes risk of intravascular injection

## Data Availability

No new data were created or analyzed in this study. Data sharing is not applicable to this article.
